# Protective efficacy of an inactivated vaccine against H9N2 avian influenza virus in ducks

**DOI:** 10.1186/s12985-015-0372-7

**Published:** 2015-09-17

**Authors:** Qiaoyang Teng, Weixia Shen, Qinfang Liu, Guangyu Rong, Lin Chen, Xuesong Li, Hongjun Chen, Jianmei Yang, Zejun Li

**Affiliations:** Innovation Team for Ecology and Virology of Animal Influenza Virus, Shanghai, 200241 China; Department of Avian Infectious Disease, Shanghai Veterinary Research Institute, Chinese Academy of Agricultural Sciences, Shanghai, 200241 China

## Abstract

**Background:**

Wild ducks play an important role in the evolution of avian influenza viruses (AIVs). Domestic ducks in China are known to carry and spread H9N2 AIVs that are thought to have contributed internal genes for the recent outbreak of zoonotic H7N9 virus. In order to protect animal and public health, an effective vaccine is urgently needed to block and prevent the spread of H9N2 virus in ducks. We developed an inactivated H9N2 vaccine (with adjuvant Montanide ISA 70VG) based on an endemic H9N2 AIV and evaluated this vaccine in ducks.

**Findings:**

The results showed that the inactivated H9N2 vaccine was able to induce a strong and fast humoral immune response in vaccinated ducks. The hemagglutination inhibition titer in the sera increased fast, and reached its peak of 12.3 log2 at 5 weeks post-vaccination in immunized birds and remained at a high level for at least 37 weeks post-vaccination. Moreover, viral shedding was completely blocked in vaccinated ducks after challenge with a homologous H9N2 AIV at both 3 and 37 weeks post-vaccination.

**Conclusions:**

The results of this study indicate that the inactivated H9N2 vaccine induces high and prolonged immune response in vaccinated ducks and are efficacious in protecting ducks from H9N2 infection.

## Findings

There is an increasing public health concern regarding the spread of H9N2 avian influenza viruses (AIVs) due to its potential for host-range extension, virulence enhancement, and providing internal genes, resulting in reassortment with other subtype influenza viruses through horizontal transmission [1–6]. The latter was exemplified by the zoonotic H7N9 virus that has caused outbreaks in China [6]. As a prominent reservoir of AIVs, ducks play an important role in the evolution, and spread of many subtypes of AIVs [7]. It was recently found that H9N2 AIVs were prevalent in domestic ducks from farms and live bird markets in China [8]; however, infections of low-pathogenic AIVs were generally overlooked, owing to the lack of clinical symptoms [6, 8]. Therefore, prevention of viral shedding of H9N2 AIVs in ducks is a challenging but crucial and important step in protecting animal and public health.

**Fig. 1:**
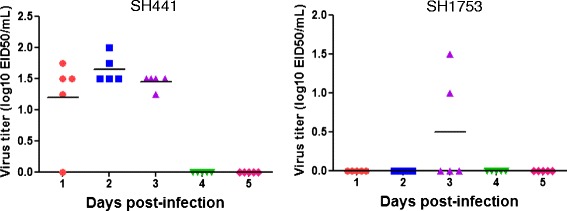
Viral titer of oropharyngeal swabs collected from ducks infected with different H9N2 viruses. Ducks were intravenously inoculated with 10^6^ EID_50_ of SH441 or SH1753. The virus titers (EID_50_) were determined for oropharyngeal swabs collected from infected ducks. The PBS-inoculated group was used as controls. The horizon line represents the mean value

**Fig. 2:**
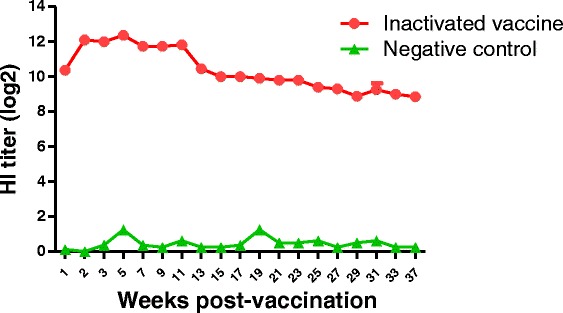
Antibody dynamics of vaccinated ducks. Six-week-old ducks were intramuscularly immunized with the developed inactivated vaccine (H9N2, SH441) The mean HI titers for sera collected weekly or bi-weekly during 37 weeks post-inoculation are shown

Vaccination has been demonstrated to be one of the most effective ways to prevent and control influenza in chickens [9]. However, research on vaccines against H9N2 AIVs in ducks is scarce. In this study, we have developed an inactivated H9N2 vaccine (with adjuvant Montanide ISA 70VG) based on an H9N2 A/duck/Shanghai/441/2009 (SH441) virus that is very closed to currently endemic H9N2 virus in China and its efficacy was evaluated in ducks.

Our preliminary studies showed that the H9N2 AIVs could not infect and replicate in ducks efficiently by an intranasal infection route. Similar results were observed for H10 subtype viruses that replicated poorly in ducks by an intranasal inoculation; however, they replicatedefficiently by the intravenous infection [10]. Therefore, we infected ducks intravenously in order to develop an H9N2 challenge model. Five H9N2 duck isolates (SH96, SH441, SH480, SH1494 and SH1753) were selected to infect groups of 9 week-old outbred sheldducks (*n* = 5). Each duck was intravenously inoculated with 10^6^ of 50 % egg infective dose (EID_50_) of each virus. Oropharyngeal and cloacal swabs were collected each day from 1 to 5 post-inoculation (dpi) for detecting virus shedding. The swab samples were used to inoculate specific pathogen free(SPF) embryonated chicken eggs and passaged twice to isolate virus. The results showed that no virus was detected in any cloacal swabs collected from all ducks inoculated with each virus. oropharyngeal swabs collected from ducks infected with SH96, SH480, or SH1494 strains were negative for virus isolation at 1–5 dpi; while the SH1753 virus was isolated from the oropharyngeal swabs collected from two of five infected ducks at 3 dpi, not at other time points [Fig. 1]. All oropharyngeal swabs collected from 5 ducks infected with the SH441 virus were positive for virus isolation at 1, 2 and 3 dpi, and the viral titers were approximately 1.5 log10 EID_50_ per ml [Fig. 1]. All these results indicate that the H9N2 AIVs do not replicate efficiently in ducks, and are consistent with previous studies in ducks and chickens [11, 12]. Wang et al. reported that the virus could be detected in the oropharyngeal swabs collected from the ducks that were intranasally infected with 10^7^ EID_50_ of different H9N2 AIVs at 2 and 3 dpi [11]. Only a small proportion of inoculated chickens shed detectable viruses found in the cloacal swabs after intranasal infection with chicken origin H9N2 viruses [12]. Moreover, 3 week-old ducks were intravenously inoculated with the above H9N2 viruses in our preliminary experiment (data not shown) and similar results were obtained as those using 9 week-old ducks in this study. This suggests that different ages of ducks show similar susceptibility to the H9N2 virus infections. Taken together, our results indicate that ducks intravenously infected with the SH441 virus can be used as a model to evaluate efficacy of the developed H9N2 inactivated vaccine.

To assess the protective efficacy of the developed H9N2 inactivated vaccine, ducks were intramuscularly vaccinated with 0.3 ml of the inactivated vaccine, and then intravenously challenged with 10^6^ EID_50_ of a homologous H9N2 (SH441) virus after 3 weeks post-vaccination. The inactivated vaccine induced a strong immune response in ducks, and the HI titers against the H9N2 virus were over 11.0 log2 after 3 weeks post-vaccination (Table 1). All five vaccinated ducks were protected against the SH441 challenge when compared to control unvaccinated birds, evidenced by the fact that no virus was recovered from the oropharyngeal swabs collected from the vaccinated birds, while virus was detected in all oropharyngeal swabs collected from the control birds.

**Table 1:** Evaluation of vaccine efficacy for protection of ducks from H9N2 viruses

Days of post-vaccination	Number of ducks	Immunization	Virus isolation from oropharyngeal swabs (mean EID_50_/ml)	
(weeks)			2 dpi	3 dpi
3	5	Vaccine	−	−
3	5	PBS	1.75	1.45
37	10	Vaccine	−	−
37	5	PBS	1.75	1.50

*dpi*, days post-infection, *PBS*, phosphate-buffered saline (negative control)

To determine dynamics of the HI antibody in immunized birds, serum was collected from H9N2-vaccinated ducks weekly or bi-weekly and tested by the HI assay. The mean HI titer increased very fast, and reached its peak at 12.3 log2 at 5 weeks post-vaccination [Fig. 2]. The HI titers were still detectable after 37 weeks post-vaccination with a higher level. In contrast, previous studies with a recombinant H5 vector vaccine showed that the highest HI titer against H5 virus only reached 7 log2 [13] and lasted for 12 weeks after post-vaccination [14]. To determine whether ducks can be protected after 37 weeks post-vaccination, a group of vaccinated ducks were challenged with a homologous H9N2 virus (Table 1). The results showed that the vaccine completely protected ducks from oropharyngeal viral shedding after challenge with the H9N2 virus even after 37 weeks post-vaccination.

In summary, our results demonstrate that the developed H9N2 inactivated vaccine is able to induce protective immune responses and is efficacious in ducks, suggesting that this vaccine could be used in poultry industry to prevent and control H9N2 influenza in ducks.

### Viruses and ducks

Five H9N2 virus isolates A/duck/Shanghai/96/2009 (SH96), A/duck/Shanghai/441/2009 (SH441), A/duck/Shanghai/480/2009 (SH480), A/duck/Shanghai/1494/2009 (SH1494), and A/duck/Shanghai/1753/2009 (SH1753) were used in this study. Each virus was amplified in 9–11-day-old SPF embryonated chicken eggs, and virus titer (EID_50_) was determined in SPF embryonated chicken eggs and calculated by the Reed and Muench method based on the HA assay of allantoic fluid of eggs inoculated with 10-fold serial dilutions of viruses [15]. Ducks (a local outbred strain of shelducks) used in this study, were confirmed to be free of any AIV infection. Before challenge, sera and swab samples were collected from each bird for the HI assay against H9 and virus isolation as described previously [16], all the results were negative. Each group of ducks were housed in a separated isolator.

### H9N2 viral infection

To test the infectivity of H9N2 viruses in ducks, groups of 9 week-old ducks were intravenously inoculated (*n* = 5) with 10^6^ EID_50_ of each H9N2 virus in a volume of 100 μL. Negative control ducks were inoculated with same volume of phosphate-buffered saline (PBS). Oropharyngeal and cloacal swabs were collected daily from 1 to 5 dpi. Oropharyngeal and cloacal swabs were incubated and treated with antibiotics to reduce bacterial contamination. The serial diluents of supernatants of oropharyngeal and cloacal swabs were inoculated into SPF embryonated chicken eggs to determine the virus titer (EID_50_) that was calculated by the Reed and Muench method.

### Vaccine development

The SH441 strain was selected for use to produce an inactivated vaccine because i) it can grow a very high titer (hemagglutination titer 12log2) and ii) it is closed related genetically and antigenically to currently endemic H9N2 viruses. To produce the inactivated vaccine, the amplified virus was inactivated by β-propiolactone (Sigma-Aldrich,St. Louis, MO, USA), then mixed with an adjuvant Montanide ISA 70VG (Seppic, Paris, France) at a ratio of 3:7 (V/V) according to the manufacturer’s instruction.

### Hemagglutination inhibition (HI) assay

Blood samples were collected weekly or bi-weekly until 37 weeks post-vaccination. Sera were isolated and HI titers were determined by HI assays as described previously [17].

### Duck vaccination and virus challenge with SH441

Six-week-old ducks were intramuscularly inoculated with 0.3 ml of the inactivated vaccine in the vaccination group (*n* = 15) or with 0.3 ml of PBS in the control group (*n* = 10). Five vaccinated and control ducks were intravenously challenged with 10^6^ EID_50_ of the SH441 virus in a volume of 100 μL at 3 weeks post-vaccination. The remaining 10 vaccinated and 5 control ducks were challenged with a same dose of virus via the same route at 37 weeks post-vaccination. Oropharyngeal swabs were collected at 2 and 3 dpi for virus detection by inoculating embryonated chicken eggs. The collected allantoic fluids were passaged twice to confirm the virus detection results.

### Ethics statement

All animal experiments described in the study (protocol number 02X09) were approved by the Animal Care and Use Committee at the Shanghai Veterinary Research Institute.

## Abbreviations

AIV: Avian influenza virus
SH96: A/duck/Shanghai/96/2009(H9N2)
SH441: A/duck/Shanghai/441/2009(H9N2)
SH480: A/duck/Shanghai/480/2009(H9N2)
SH1494: A/duck/Shanghai/1494/2009(H9N2)
SH1753: A/duck/Shanghai/1753/2009(H9N2)
PBS: Phosphate-buffered saline
SPF: Specific-pathogen free
HA: Hemagglutination
dpi: Day post-inculation
EID_50_: The 50 % egg infective dose
HI: Hemagglutination inhibition .
